# Case Report: A Novel Activating FLT3 Mutation in Acute Myeloid Leukemia

**DOI:** 10.3389/fonc.2021.728613

**Published:** 2021-09-30

**Authors:** Samantha Bruno, Lorenza Bandini, Agnese Patuelli, Valentina Robustelli, Claudia Venturi, Manuela Mancini, Dorian Forte, Sara De Santis, Cecilia Monaldi, Alessandra Grassi, Gabriella Chiurumbolo, Stefania Paolini, Gianluca Cristiano, Cristina Papayannidis, Chiara Sartor, Jacopo Nanni, Emanuela Ottaviani, Antonio Curti, Michele Cavo, Simona Soverini

**Affiliations:** ^1^ Department of Experimental, Diagnostic and Specialty Medicine, University of Bologna, Bologna, Italy; ^2^ Istituto di Ricovero e Cura a Carattere Scientifico (IRCCS) Azienda Ospedaliero-Universitaria di Bologna, Istituto di Ematologia “Seràgnoli”, Bologna, Italy

**Keywords:** acute myeloid leukemia, FLT3 mutation, NGS - next generation sequencing, targeted therapy, midostaurin

## Abstract

FMS-like tyrosine kinase 3 (FLT3) is among the most common driver genes recurrently mutated in acute myeloid leukemia (AML), accounting for approximately 30% of cases. Activating mutations of the FLT3 receptor include internal tandem duplications (ITD) that map to the auto-inhibitory juxtamembrane (JM) domain or point mutations within the tyrosine kinase domain (TKD). Several FLT3 tyrosine kinase inhibitors have been developed in the last few years, but midostaurin is currently the only one approved for the treatment of newly diagnosed patients harboring *FLT3* mutations. Here we describe for the first time a novel in-frame deletion in exon 14 (JM domain) of the *FLT3* gene, that we identified in a young woman with CBFb-MYH11-positive AML. We demonstrated that this novel *FLT3* variant is pathogenic, since it is responsible for constitutive activation of FLT3 receptor. Finally, *ex-vivo* studies demonstrated that this novel mutation is sensitive to midostaurin.

## Introduction

Acute myeloid leukemia (AML) is a highly heterogeneous haematological malignancy characterized by a wide range of genomic alterations responsible of defective regulation of the differentiation and self-renewal programs of hematopoietic stem cells. FMS-like tyrosine kinase 3 (FLT3) is a member of class III receptor tyrosine kinases that exhibits activating mutations in approximately 30% of AML patients, making it one of the most recurrently mutated genes (1, 2). Notably, there are two types of *FLT3* mutations which include the internal tandem duplications (ITD) within the auto-inhibitory juxtamembrane (JM) domain, described in about 25-30% of AML patients, and the less frequent gain-of-function mutations occurring in about 8% of AML cases as point mutations in the activation loop of the tyrosine kinase domain (TKD) (1, 2). The leukemogenic relevance and inferior overall survival of *FLT3-ITD* mutations have been shown, although it has been demonstrated that the prognostic impact is affected by both mutant allele burden and presence of co-existing mutations (3, 4). Indeed, high allele ratio (AR>0.5; FLT3-ITD^high^) is associated with higher disease risk, whereas low AR (<0.5; FLT3-ITD^low^) is associated with intermediate risk, which becomes favourable in case of co-occurrence of the nucleophosmin 1 (NPM1) mutation (4). Concerning the *FLT3-TKD* mutations, their prognostic significance still remains unclear, with numerous conflicting data (3, 5–8). The clinical impact of ITD and TKD mutations of *FLT3* is dependent on the subsequent constitutive activation of the tyrosine kinase, that activates its downstream signalling pathways including the signal transducer and activator of transcription 5 (STAT5), RAS/mitogen-activated protein kinase (MAPK)/extracellular-signal-regulated kinase (ERK), phosphoinositide 3-kinase (PI3K)/Serine-Threonine Kinase 1 (AKT) (9–13). These targets have been associated with increased proliferation and survival of the leukemic cell population (14). In light of the crucial pathogenetic role of FLT3 constitutive activation, small molecule inhibitors have been developed as new promising therapies to treat this subset of AML patients.

Here we describe for the first time a novel *FLT3* in-frame deletion and its impact on downstream signalling pathways showing the activation effect of such mutation on FLT3 receptor. Moreover, *ex-vivo* study performed on mutated primary cells sought to demonstrated their sensitivity to midostaurin, a multi-targeted kinase inhibitor active against FLT3, currently approved for first-line treatment of *FLT3*-mutated AML (15).

## Case Description

### Clinical Case

**Figure 1:**
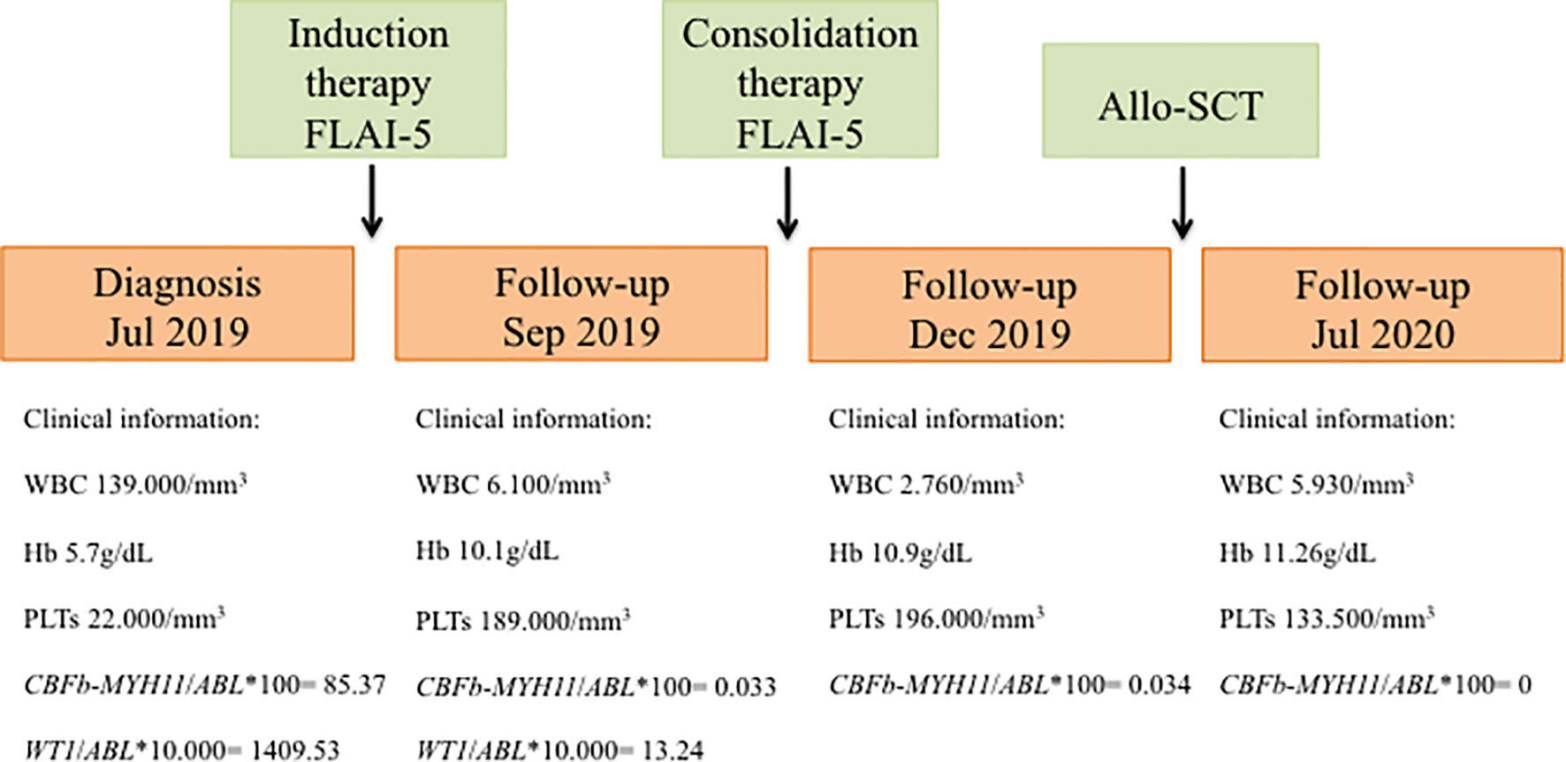
Schematic representation showing timeline of treatment course and clinical information. FLAI-5, fludarabine-cytarabine-idarubicin; Allo-SCT, allogeneic stem cell transplantation; WBC, white blood cells; Hb, hemoglobin; PLTs, platelets.

A 32 years-old woman which presented with persistent fever associated with marked hyperleukocytosis [white blood cells (WBC), 319,000/mm^3^], anemia [hemoglobin (Hb), 5.7 g/dL] and thrombocytopenia [platelets (PLTs), 22,000/mm^3^] was admitted to our hospital in July 2019. Morphological analysis of bone marrow (BM) aspirate suggested a diagnosis of acute myelomonocytic leukemia with 80% of myeloid blasts. Computed tomography (TC) examination of the brain detected central nervous system (CNS) localization of blast cells. Immunophenotypic analysis of leukemic cells from the BM aspirate confirmed the expression of several markers of hematopoietic stem and progenitor cells, including CD34, CD13, CD33, CD117, MPO, CD14, CD64, and aberrant expression of CD4. Karyotype and fluorescence *in situ* hybridization (FISH) analyses of BM nuclei identified the inv(16)(p13;q22) chromosome rearrangement in 20/20 metaphases examined. This was confirmed by quantitative RT-PCR that detected the presence of *CBFβ-MYH11* transcript type A (*CBFβ-MYH11*/ABL*100 = 85.37), along with over-expressed *WT1* (ratio *WT1*/ABL*10,000 = 1409.53). Therefore, the patient was diagnosed with M4 inv(16) acute myeloid leukemia (AML) according to French-American-British (FAB) classification and was classified as favourable cytogenetic risk based on cytogenetic and molecular biology data according to ELN 2017 (4). However, in light of CNS localization at diagnosis, the patient was considered at high risk of relapse. A FLAI-5 (fludarabine-cytarabine-idarubicin) regimen was selected as induction chemotherapy, which resulted effective and well tolerated. The follow-up evaluation performed after 2 months of induction therapy showed a good response: the patient exhibited a peripheral blood count with WBC levels of 6,100/mm^3^, Hb levels of 10.1 g/dL and PLTs of 189,000/mm^3^. Accordingly, BM evaluation by morphology, immunophenotyping and molecular biology revealed complete remission (CR; *CBFβ-MYH11*/ABL*100 = 0.0332; *WT1*/ABL*10,000 = 13.24) and TC of the brain was negative for SNC localization of blast cells. Therefore, the patient continued with 1 cycle of consolidation therapy with the FLAI-5 regimen. As reported above, due to CNS localization at diagnosis, the patient underwent allogeneic stem cell transplantation (allo-SCT) receiving haploidentical peripheral blood stem cells from her brother. After transplant, she remained in complete remission until the last follow-up. **Figure 1** summarizes relevant clinical course and diagnostic results.

## Detection of a Novel FLT3 Deletion and Investigation of Its Potential Role

**Figure 2:**
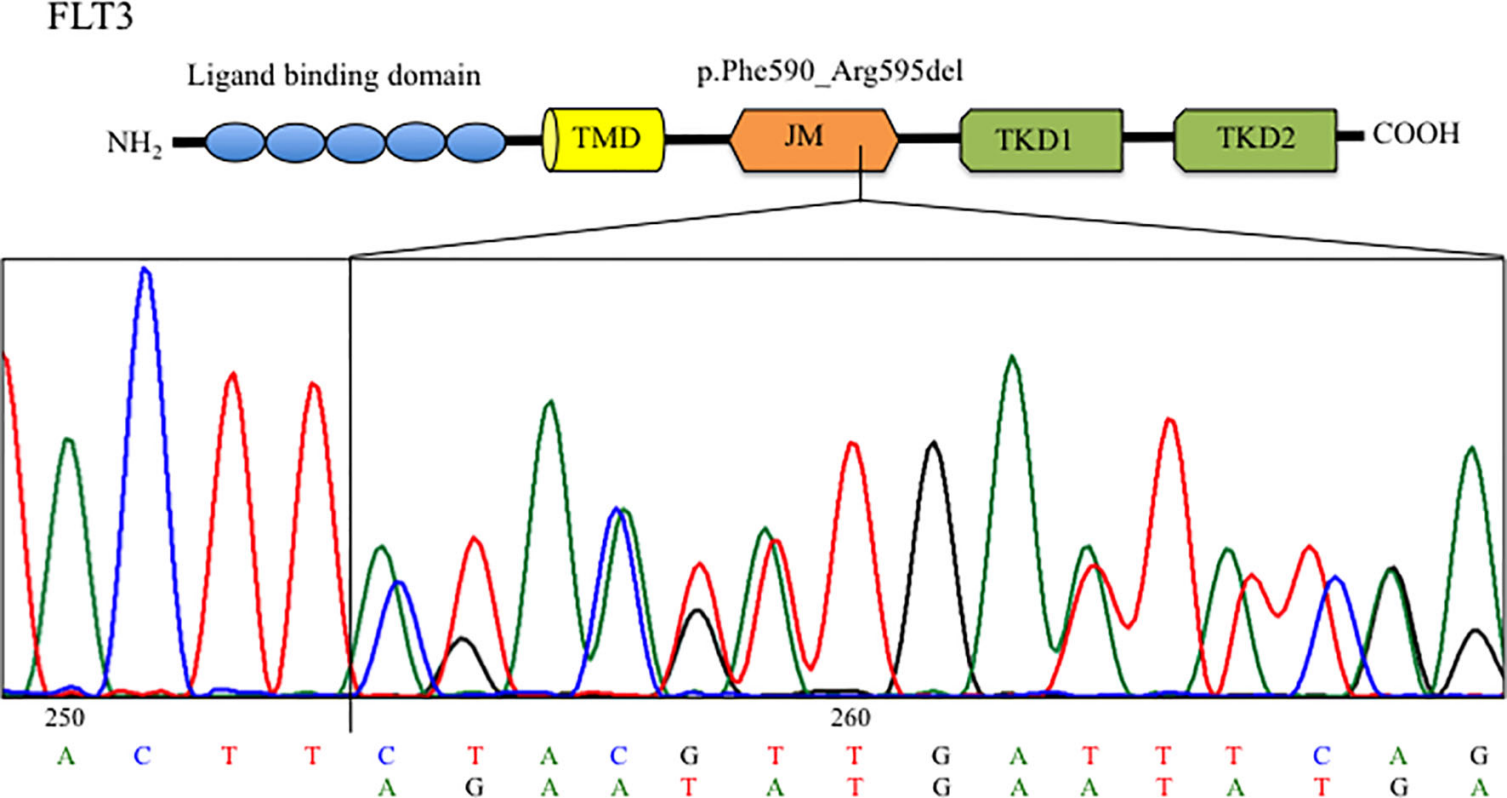
Sanger sequencing chromatogram showing the FLT3 deletion identified in the BM at diagnosis. TMD, transmernbrane domain; JMD, juxtamembrane domain; TKD, tyrosine kinase domain.

In light of the clinical utility of next-generation sequencing (NGS) for the assessment of the genetic landscape of AML providing diagnostic and prognostic information, the AmpliSeq Myeloid Panel (Thermofisher) was performed on primary blast cells enriched by density gradient centrifugation from the patient bone marrow sample collected at diagnosis. NGS analysis revealed a *FLT3* mutation with a variant allele frequency (VAF) of 40%, represented by an in-frame deletion at exon 14 of the JM domain (c.1770_1784del15; p.Phe590_Arg595delinsLeu). Interrogation of the COSMIC database indicated that this deletion had never been reported before. The mutation was confirmed by Sanger sequencing (**Figure 2**) and mutant-to-wild-type allelic ratio (AR) was obtained through PCR-electrophoresis and fragment analysis (*FLT3-ITD*-AR=1.1). The *FLT3* mutation became undetectable in the sample collected at the time of CR after induction chemotherapy.

**Figure 3:**
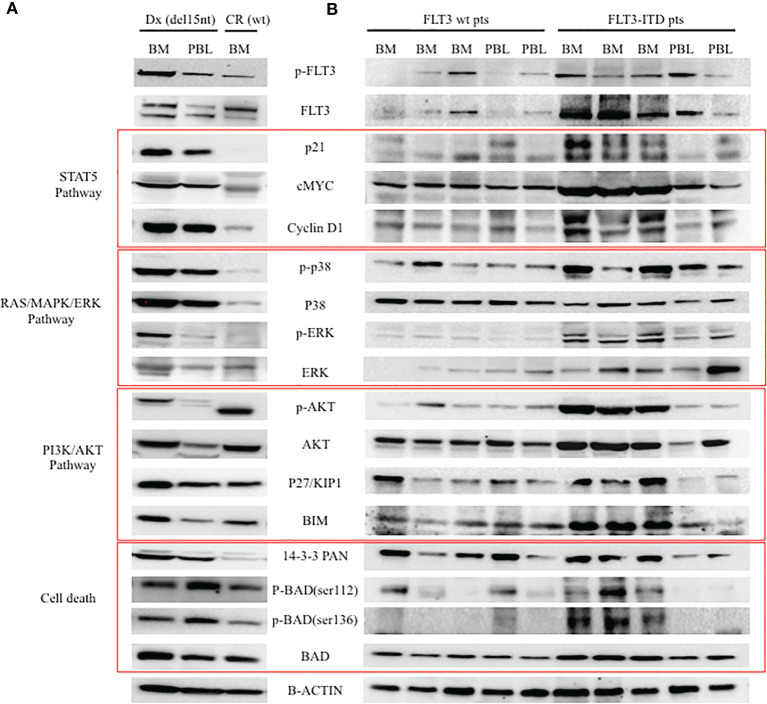
Western blot analysis of FLT-3 expression and of its down-stream signalling pathway. **(A)** Analysis of patient cells at two different time points (Dx: diagnosis with FLT3 carrying the c.1770_1784del15; CR: complete remission with wt FLT3). **(B)** Analysis of 5 AML patients with wt FLT3 along with 5 patients carrying FLT3-ITD, used as control of FLT3 downstream signalling. BM, bone marrow; PBL, peripheral blood.

**Figure 4:**
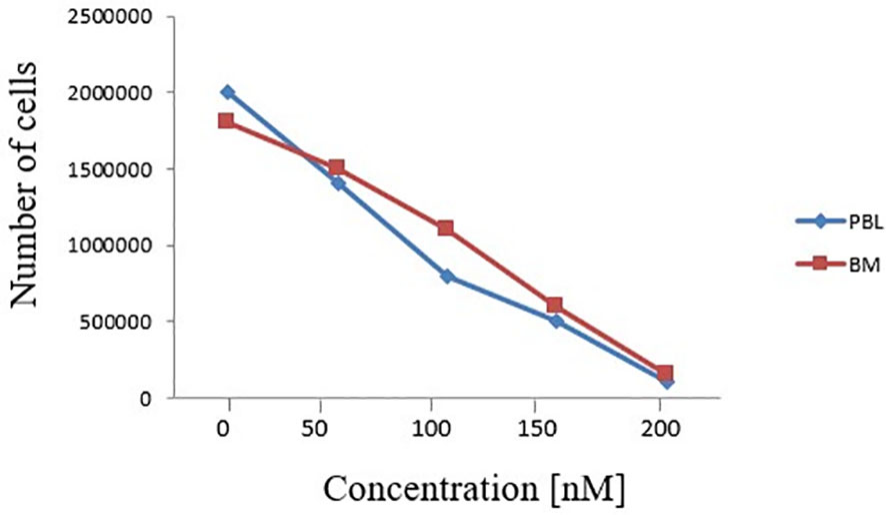
Effects of increasing doses of midostaurin on the clonogenic capacity of primary BM and PBL cells carrying the reported FLT3 deletion (c.1770_1784del15; p.Phe590_Arg595delinsLeu).

In order to understand whether the newly described FLT3 alteration influenced its cellular activity, we analysed by Western blot (WB) the expression and activation of some target proteins downstream of FLT3. As shown in **Figure 3A**, we observed that the deletion in the JM domain resulted in the constitutive activation of FLT3, with the sample at diagnosis showing increased levels of phosphorylated FLT3 and of its downstream signalling molecules. Five AML patients with wild-type (wt) *FLT-3* and 5 AML patients carrying canonical *FLT3-ITD* mutations (with an AR ranging from 0.8 to 1.5) were used as control for protein expression levels of FLT3 downstream signalling molecules (**Figure 3B**). In particular, the sample at diagnosis, showed increased levels of p21, c-MYC and Cyclin D1, that were associated with the activation of STAT5 signalling pathway, together with the up-regulation of MAPK-activated proteins p-ERK and p38. In addition, the activation of FLT3 induced p27 and BIM expression, which are regulated by AKT and FOXO3A signalling, as well as the up-regulation of 14-3-3 and phosphorylated BAD (serine 112 and 136) that are responsible for the block of cell death. Results were superimposable to what we could observe in the 5 *FLT3-ITD* mutated samples as compared to *wt FLT3* samples. As expected, the sample at remission showed a significant reduction of phosphorylated FLT3 and of the activation of all the above mentioned targets, supporting the direct link between activated signalling molecules and the observed FLT3 mutation. Lastly, to investigate the clinical relevance of the described mutation, we assessed the effects of midostaurin on the clonogenic potential of leukemic blasts cells isolated from the patient at diagnosis. Midostaurin induced a dose-dependent reduction in colony formation with an LD_50_ of 100nM, suggesting that the newly reported mutation is sensitive to midostaurin inhibition (**Figure 4**).

## Conclusion and Discussion

*FLT3* is the most frequently mutated gene in AML patients, with a strong disease specificity (2), and encodes is one of the key molecules involved in the pathogenesis of AML. Indeed, *FLT3* is one of the markers currently used for the molecular genetic diagnosis and risk stratification of AML (4). The clinical relevance of ITD and TKD mutations in AML pathogenesis and progression highlighted the need for specific compounds targeting FLT3 constitutive activation. Consequently, during the last years several inhibitors have been developed as target therapies to improve the outcome of *FLT3*-mutated patients (16, 17). In this study, we describe for the first time a novel *FLT3* mutation identified in an AML patient, consisting in a deletion of 15 nucleotides in the JM domain, where the *FLT3*-ITDs usually localize. Interestingly, other case reports showing noncanonical *FLT3* deletions have been published, and all these mutations cluster in the JM domain, a highly conserved region that mediates the activation of the FLT3 receptor (18–20). There are limited functional data for deletions in the JM domain, however existing knowledge suggest that any type of mutation, including deletions, in the JM sequence could potentially activate the FLT3 tyrosine kinase receptor, with consequent pathogenetic and clinical impact. In this study, we demonstrated by WB that the newly reported deletion indeed induces the constitutive activation of FLT3 and of its downstream effectors, including STAT5, ERK and AKT pathways. The patient showed a good response to conventional chemotherapy and underwent transplant. However, we also evaluated by *ex-vivo* treatment the efficacy of midostaurin, the TKI currently approved for the treatment of *de novo FLT3*-mutated patients, finding that midostaurin could effectively reduce the clonogenic capacity of primary blast cells. Although additional studies are needed to better characterize the functional consequences and TKI sensitivity of this deletion, our results are corroborated by recent work from Young and colleagues. They used a scanning mutagenesis approach to generated a panel of FLT3 deletions (clustering in the region comprised between K568 to V581) demonstrating that this class of mutations lead to the activation of signalling molecules downstream to FLT3 and are targetable with several TKI inhibitors (20). In conclusion, our results suggest the clinical relevance of the novel *FLT3* deletion herein reported, that acts as a standard activating ITD mutation and can be successfully targeted by midostaurin. Our findings provide useful information for clinicians treating AML patients, highlighting the importance to characterize and monitor the genomic alterations of AML patients.

## Data Availability Statement

The original contributions presented in the study are included in the article/supplementary materials. Further inquiries can be directed to the corresponding author.

## Ethics Statement

The studies involving human participants were reviewed and approved by local Ethics Committee (protocol 112/2014/U/Tess of Policlinico Sant’Orsola-Malpighi). The patients/participants provided their written informed consent to participate in this study.

## Author Contributions 

SB, LB, DF, SS, EO and MM contributed to the conception, research design, drafting of the manuscript, and conceptualization methodology. LB and AP performed NGS analysis. VR, CV, SDS, and CM performed sanger sequencing and PCR-electrophoresis and fragment analysis. GaC and AG performed FISH analysis. SB and MM performed WB analysis. SB and DF performed colony-formation assay. SP, GiC, CP, CS, JN, AC, and MC provided clinical data and enrolled the patient for the study. SS and AC reviewed and edited the manuscript. EO, SS, and MC acquired the funding. MM, EO and SS provided the supervision of the study. All authors contributed to the article and approved the submitted version.

## Conflict of Interest 

AC was employed by Novartis, Pfizer, Abbvie and acted as speaker in Advisory Board for Novartis and Abbvie. CP was employed by Astellas, Amgen and acted as speaker in Advisory Board for Abbvie, Janssen, Novartis, Pfizer and Astellas.

The remaining authors declare that the research was conducted in the absence of any commercial or financial relationships that could be construed as a potential conflict of interest.

## Publisher’s Note

All claims expressed in this article are solely those of the authors and do not necessarily represent those of their affiliated organizations, or those of the publisher, the editors and the reviewers. Any product that may be evaluated in this article, or claim that may be made by its manufacturer, is not guaranteed or endorsed by the publisher.
